# Reviews and Book Notices

**Published:** 1880-08

**Authors:** 


### Reviews and Book Notices.

ARTICLE XI.—A SYSTEM OF MEDICINE. Edited by J. Russell Reynolds, M.D., F.R.S., Fellow of the Royal College of Physicians of London; Fellow of the Imperial Leopold-Carolina Academy of Germany; Fellow of University College, London; Professor of the Principles and Practice of Medicine in University College; Physician to University College Hospital; Examiner in Medicine to the University of London. With numerous additions and illustrations, by Henry Hartshorne, A.M., M.D., Fellow of the College of Physicians of Philadelphia; formerly Professor of Practice of Medicine in Medical Department of Pennsylvania College, and Physician to the Episcopal Hospital of Philadelphia; lately Professor of Hygiene in the University of Pennsylvania, and Professor of Hygiene and Diseases of Children in the Woman's Medical College of Philadelphia, etc., etc. In three volumes. Philadelphia: Henry C. Lea. 1879.

This is a voluminous work, comprised in three large volumes of about eleven hundred pages each, published in the usual good style of the well-known publishing house of Henry C. Lea. It consists largely of a republication of a work that originally appeared several years since, made up of articles from a large number of eminent English writers, and edited by J. Russell Reynolds, M.D., of London, England. The present work is not a new edition of the original, revised by the several authors contributing to it, for many of those are dead; but a republication of the original text, with notes by the English editor, and still more numerous and important notes and additions by the American editor, Dr. Henry Hartshorne, of Philadelphia. It embraces articles from no less than sixty-five contributors on as many different diseases or groups of disease. The English editor classifies diseases primarily into general and local. The first are such as involve disturbance of all the functions of the system, and the second such as are located in some structure or organ primarily, and induce general disturbance only as a secondary result.

The first volume contains an introductory article by Dr. Reynolds, and the several papers on general diseases, and also those on diseases of the nervous system. The general diseases are divided into, first, "those determined by agents operating from without;" and, second, "those determined by conditions existing within the human body." In the first division are included influenza, whooping-cough, diphtheria, scarlet fever, dengue, roseola, measles, rötheln, parotitis, sudamina and miliaria, varicella, small-pox, vaccination, glanders, hydrophobia, enteric or typhoid fever, typhus fever, relapsing fever, yellow fever, epidemic cerebro-spinal meningitis, the plague, erysipelas, pyæmia, malarial fevers, dysentery, epidemic cholera, and constitutional syphilis. In the second division are included scorbutus, purpura, chlorosis, rickets, scrofula, gout, rheumatoid arthritis, rheumatism and gonorrhœal rheumatism. Three of the diseases just mentioned—namely: rötheln or German measles, scrofula and chlorosis—have been added by the American editor. The essays on those diseases termed general, occupy the first 579 pages of the first volume of the work. The next 500 pages, completing the volume, are occupied with the consideration of affections located in the nervous system. In this part are found insanity, hypochondriasis, hysteria, ecstasy, hystero-epilepsy, catalepsy, somnambulism, sunstroke, alcoholism, vertigo, chorea, paralysis agitans, athetosis, writers' cramp, convulsions, epilepsy, muscular anæsthesia, wasting palsy, metallic tremor, simple meningitis, tubercular meningitis, chronic hydrocephalus, meningeal hæmorrhage, congestion of the brain, cerebritis, softening of the brain, adventitious products of the brain, cerebral hæmorrhage and apoplexy, abscess of the brain, spinal meningitis, myelitis, congestion, tetanus, locomotor ataxy, neuritis and neuroma, neuralgia, local paralysis from nerve disease, local spasm, torticollis, and local anæsthesia.

The second volume, containing 935 pages, is occupied with the consideration of the diseases of the respiratory and circulatory systems of organs. Diseases of the Larynx are treated by Morrell Mackenzie, M.D.; Croup, by William Squire, L.R.P.C.; Emphysema of the Lungs, by Sir William Jenner; Asthma, by Hyde Salter, M.D., F.R.S.; Phthisis Pulmonalis, by J. Hughes Bennett, M.D., F.R.S.; Cancer of the Lungs, by Herman Beigel, M.D.; Pneumonia, Syphilitic Affections of the Lungs, and Brown Induration of the Lung, by Wilson Fox, M.D., F.R.C.P.; Cirrhosis of the Lung, by H. Charlton Bastian, M.D., F.R.S.; Apneumatosis, by George Hewitt, M.D., F.R.C.P.; Bronchitis, by Frederick T. Roberts, M.D.; Pleurodynia, Pleurisy, Hydrothorax and Pneumothorax, by Francis E. Anstie, M.D., F.R.C.P.; On the Weight and Size of the Heart, Lateral or Partial Aneurism of the Heart, and on Adventitious Products in the Heart, by Thomas B. Peacock, M.D., F.R.C.P.; On the Position and Form of the Heart and Great Vessels, Pericarditis, Adherent Pericardium, and Endocarditis, by Francis Sibson, M.D., F.R.S.; Pneumo-Pericardium and Hydro-Pericardium, by J. Warburton Begbie, M.D.; Angina Pectoris and Allied States, by Prof. Gairdner, M.D.; Carditis, Atrophy of the Heart, Hypertrophy, Dilatation, Fatty Diseases, and Fibroid Diseases of the Heart, by W. R. Gowers, M.D.; Diseases of the Valves of the Heart, by C. Hilton Fagge, M.D., F.R.C.P.; Mediastinal Tumor, Diseases of the Aorta, Aneurism of the Thoracic Aorta, Diseases of the Pulmonary Artery, and Diseases of the Coronary Arteries, by R. Douglas Powell, M.D., F.R.C.P.; Aneurism of the Abdominal Aorta, by William Murray, M.D., F.R.C.P.; Diseases of the Arteries and Veins, Cardiac Concretions, Thrombosis and Embolia, by John Syer Bristowe, M.D,, F.R.C.P.; Hæmophilia, by Henry Hartshorne, A.M., M.D.; and Inflammation of the Lymphatic Vessels, by J. Russell Reynolds, M.D., F.R.S.

The third volume comprising nearly 1000 pages, treats of the "Diseases of the Digestive, Blood-Glandular, Urinary, Reproductive, and Cutaneous systems." The contributions on Diseases of the Stomach, by Wilson Fox, M.D., F.R.S.; Diseases of the Mouth, Fauces, Pharynx, and Œsophagus, by Charles E. Squarey, M.B.; Enteralgia, Peritonitis, Diseases of the Spleen and Pancreas, by John Richard Wardell, M.D., F.R.C.P.; Enteritis, Obstruction of the Bowels, Ulceration of the Bowels, Cancerous and other growths of the Intestines, Diseases of the Cæcum and Apendix Vermiformis, Tubercle of the Peritoneum, Carcinoma of the Peritoneum, Affections of the Abdominal Lymphatic Glands, and Ascites, by John Syer Bristowe, M.D., F.R.C.P.; Colic, Colitis, Dysentery, Fatty Liver, Cancer of the Liver, Hydatid Disease of the Liver, and Waxy Disease of the Liver, by J. Warburton Begbie, M.D., F.R.C.P.E.; Diarrhœa, Jaundice, Biliary Calculi, Chronic Atrophy of the Liver, and Acute or Yellow Atrophy of the Liver, by Edward Goodeve, M.B.; Diseases of the Rectum and Anus, by Thomas Blizard Curling, F.R.S.; Intestinal Worms, by W. H. Ransom, M.D.; Hepatalgia, by Francis Edward Anstie, M.D., F.R.C.P.; Congestion of the Liver, Suppurative Inflammation, and Gangrenous Inflammation of the Liver, by W. C. Maclean, M.D.; Splenic Leucocythæmia, Hodgkin's Disease, by William R. Gowers, M.D.; Addison's Disease, by Samuel Wilks, M.D., F.R.S.; Exophthalmic Goitre, by Hermann Beigel, M.D.; Diabetes Mellitus, and Diabetes Insipidus, by T. Lauder Brunton, M.D., F.R.S.; Nephralgia, by W. R. Basham, M.D., F.R.C.P.; Hæmaturia, Endemic Hæmaturia, Hæmatinuria and Paroxysmal Hæmatinuria, Albuminuria, Congestion of the Kidneys, Bright's Disease, Anomalies of Form, Position, and Number of the Kidneys, by William Roberts, M.D., F.R.S.; Diseases of the Renal Bloodvessels, Calculous Disease of the Kidney, Hydronephrosis, Renal Abscess, Tumors and new growths of the Kidney, and Diseases of the Urethra, by Frederick T. Roberts, M.D., F.R.C.P.; Nephritis and Pyelitis consecutive to Affections of the Lower Urinary Tract, by Marcus Beck, M.S.; Affections of the Bladder, by Sir Henry Thompson, M.B., F.R.C.S.; Changes in the Shape and Position of the Uterus, and Disorders of Uterine Functions, by Graily Hewitt, M.D., F.R.C.P.; Inflammation of the Uterus, Metritis, Pelvic Hæmatocele, and Pelvic Cellulitis, Pelvic Peritonitis, by William Overend Priestly, M.D., F.R.C.P.; Growths in the Uterus, Inflammation of the Ovary, and Ovarian Tumors, by John Williams, M.D.; Cutaneous Diseases, by J. Balmanno Squire, M.B.; to which are added Cholera Morbus, Cholera Infantum, Trichina Spiralis, Bronchocele, Progressive Pernicious Anæmia, and Spermatorrhœa, by Henry Hartshorne, A.M., M.D.

We have given the foregoing detail of the contents of these volumes that our readers may judge more correctly of the magnitude of the work. The careful reader will note some duplications and incongruities. For instance dysentery is classed as a general disease and treated in the first volume by Dr. Meacham, and also as a local disease and treated in the third volume by Dr. J. Warburton Begbie. The same is true of roseola. Rather curiously, sudamina and miliaria are assigned a place in the first volume with the *general diseases*, and gravely treated in a short article by Dr. Sidney Ringer. Yet this writer expressly states that these eruptions "are characteristic of no particular disease, but are produced by sweating; and hence they often occur on the decline of fevers, and especially on those critical days when the sweating is most profuse." It would appear to us quite as appropriate to call the *rose* spots of typhoid fever, or any other mere *symptom*, a general disease, as to apply that title to sudamina aud miliaria. These, however, are minor matters. The greater defects in the work arise from the fact, that a large number of the articles treating of the most important topics were written some fifteen years since, and at a time when the so-called stimulating or alcoholic treatment of general fevers, and almost all acute diseases, was at its height in Great Britain; and before the antipyretic doctrines of the present day had attracted very general attention. The attempt to supply these defects by the short notes of the American editor, is only partially successful, and often seems to make the contrasts and discrepancies more prominent. For instance, the article on typhoid fever was written by Dr. John Harley, of London, in 1865, and besides being fifteen years behind the present state of medical progress, it contains a number of paragraphs much better calculated to lead the reader into serious errors, both of pathology and of therapeutics, than to aid him to correct views in regard to this important disease. Here is the first paragraph under the head of Pathology. "If we carefully regard the incipient symptoms of enteric fever, we shall find that they have reference to derangement of the hepatic function. Often, long before the graver symptoms are developed, the patient loses appetite, the bowels are constipated, and the stools pale; the tongue is foul, and the digestion much impaired. All these symptoms point to a defective secretion of bile, and to a state of approaching inanition. Such a torpid condition of the liver may be produced in two ways in the development of enteric fever. It may result from severe or prolonged vascular congestion, in which the other internal organs participate, or it may be the effect of some morbific agent, carried by the portal vein from the intestinal surface into the liver, and causing, by a direct action upon its secreting corpuscles, derangement, or more or less complete paralysis, of its functions." (See Vol. I, p. 218). Again, he says: "Primary vascular congestion of the liver, no matter how produced, leads to a vitiation of the secretions of the alimentary canal; nervous exhaustion results from arrested nutrition. Under these conditions the liver begins to degenerate, and the intestinal mucous membrane tends to ulcerate, the blood is imperfectly depurated, and general febrile disturbance ensues." (P. 219). Still pursuing the same subject, he adds: "Not the least important function of the liver, is, to prevent by its antiseptic properties the decomposition of the chyme; take away this preservative influence altogether from the system, and fermentation with the escape of gas and tymanitic distention follow. The impure chyme irritates the debilitated and congested mucous membrane, and what wonder then if inflammation, ending in ulceration of Peyer's patches and the follicular glands, should result." Finally, he says: "From the foregoing observations it will be seen that we are induced to conclude that the disease arises from vitiation of only a portion of the venous blood, and that the constitutional symptoms are in many cases, due to consequent derangement of the hepatic function."

By these paragraphs, and still more by the whole text from which they are taken, it is seen that Dr. Harley comes as near placing the essential pathology of typhoid fever in the *liver* as Broussais did in the gastro-enteric mucous membrane, or Clutterbuck in the brain. How can he reconcile his supposed primary hepetic derangement and degeneration, and consequent decomposition of chyme as primary pathological changes, causative of both the general fever and the local intestinal lesions, with the well known fact that post mortem examinations show less morbid changes in the duodenum and upper part of the jegunum, where the supposed chyme decomposition would take place, than in any other part of the alimentary canal; and with the further fact that the liver very generally shows much less change either in size or structure than either the spleen or the mesenteric glands. His idea, as expressed in the last pharagraph, that the mischief arises from a *vitiation* of only "a portion of the venous blood," seems to be based on the assumption that all the morbid material taken up from the intestinal surfaces and carried into the portal vein necessarily went directly into the liver. But inasmuch as a large part of the blood in the portal vein passes directly into the vena cava without entering any part of the liver, it is a little difficult to conceive by what peculiar mode of separation all the vitiating material is kept in just that part of the common mass of blood in the portal vessels that enters the branches ramifying in the liver without passing in equal proportion in that part going directly into the vena cava. It is hardly necessary to comment further on these crude and fanciful pathological notions of Dr. Harley. And yet they exert an important influence over his views of treatment, as may be seen by the following paragraph from page 247. In reference to treatment he says: "The indications in the early period of the disease are to relieve internal congestion, and to revive the function of the liver." And after this early stage is passed, he says: "In the further progress of the disease, our treatment will have almost exclusive reference to the abdominal lesion."

It is not necessary to pursue the examination of this article further to make good our assertion that its doctrines are crude and in some respects obsolete; and the same is true of many other articles in relation to some of the most important diseases the practitioner is called upon to treat. What has been pointed out, however, will indicate to our readers the nature of our objections to this great work. Yet we are not willing to let even the introductory chapter by Dr. Reynolds pass without a protest against one or two of its plausible vagaries. For instance on the first page, in discussing a definition of disease, he says: "Disease is a condition of the individual man; it is always something more than the changes that we yet can recognize and describe in any particular organ or its functions. It is the *man* who is ill; and, under all circumstances of illness, he has a *diminished life*." Admit that "it is the *man* who is ill," what have we gained by the admission? Can we have any conception of the *man*, whether *ill* or not, except as represented by the aggregation of his physical organs and mental faculties? Or can we form any conception of *life* in our present state of existence except as represented by the organs and structures of the body in a state of activity? Is *life* an abstract entity consisting of a given amount of something distinct from the organs or physical structures, capable of increase by health and diminution by disease? To talk about the *man* being ill, and the *illness* being always a *diminished life*, in any other sense than as indicated by the conditions of the structures and functions which unite to make the *man*, is to amuse ourselves with incomprehensible figures of speech, full of sound, but signifying nothing. But starting with such ideas of disease, we are not surprised to find him closing the same chapter with the following therapeutic law: "In all treatment, therefore, what is *general* is to be dealt with upon the basis of a true appreciation of the general pathological condition, and this in spite of all theories in regard of local changes, however they may be termed, whether they come to us with names hoary with age, or scarcely intelligible, and sometimes even ludicrous from their novelty. If the general condition be one of weakness, it matters not that the brain, the heart, or the lungs may be in a state of so-called 'inflammation', the *weakness* is the *one thing* that demands immediate treatment, and to neglect its treatment is to run the risk of sacrificing the patient to a theory of a compound state even now but imperfectly understood. This is the starting point, the essential element in therapeutics," etc. In other words we are here taught that all disease is a diminution of a life. A condition of diminished life must be one of general weakness, and this general weakness is the *one thing* demanding immediate treatment, without regard to local conditions of the brain, heart, lungs, or other structures. We can hardly conceive of a doctrine more unphilosophical than this; or one more dangerous to the patient when practically applied in the treatment of diseases. Having briefly indicated what we deem objectionable in the volumes, with examples sufficient to indicate their nature, we wish to do justice both to our readers and the publisher by saying that these three ponderous volumes contain a vast amount of valuable information. While they are too voluminous, and contain too much matter that is obsolete, or not in harmony with the present state of medical knowledge to be fit for use as text-books for medical students, yet as works of reference in the library of the discriminating practitioner, the medical investigator, and the medical teacher, they are of real value. D.

ARTICLE XII.—LECTURES ON THE HUMAN EYE IN ITS NORMAL AND PATHOLOGICAL CONDITIONS. By Adolph Alt, M.D., Lecturer on Ophthalmology and Otology in Trinity Medical School, Canada. With ninety-five illustrations by the author. G. P. Putnam's Sons, New York.

In his preface to this work the author says: "A book treating systematically on this subject, and especially on the histological conditions of the pathological human eye, whose execution and price would allow it to be of usefulness to everyone interested in the matter, has been to this day a want often felt.

Dr. Alt is to be congratulated upon the very efficient manner in which he has supplied this want. The superior advantages for study, in this line, which the doctor has enjoyed have been well improved. This book is confined entirely to the eye-ball, and while it contains only a little more than 200 pages, it represents an immense amount of labor. The normal anatomy is concisely and clearly presented, but this we may find in a number of the text books on diseases of the eye. The chief value of the work lies in the full and special attention given to the pathological anatomy of the different structures of the globe. The numerous wood cuts from drawings by the author add very much to the value of the book, and reflect credit both upon the author and publishers.

It is very much to be regretted that this excellent little book should be marred by so many trifling errors. Comeo-scleral is comea-scleral in a number of places; protoplasmatic is protaplasmatic, lymphatic is lympathic, peripheral is periphecal, dependent is dependant, serous is serious, etc. A number of sentences are not as clear as they might be. Page 8.: "The fibers into which *Bowman's* layer, is, split up and the anterior lamellæ of the corneal parenchyma together enter the subconjunctival tissue in such a way that these two parts cannot be separated," etc. Page 14.: "This is, however, always the case when infiltration results, goes in the purulent form of knatitis with partial destruction of the corneal tissue." Again, on page 203: "Some authors have seen transverse striation on these fibers, and therefore considered them to be of a character muscular cells sometimes found between them, are wandering (lymphatic) cells." But these errors do not seriously impair the value of the book, and can be elided when a second edition appears, for which we will not probably have long to wait.

In our opinion this little book is a valuable addition to the literature of the subject upon which it treats. W. T. M.

ARTICLE XIII.—ROCKY MOUNTAIN HEALTH RESORTS. An Analytical Study of High Altitudes in Relation to the Arrest of Chronic Pulmonary Disease. By Charles Denison, A.M., M.D.

The author of this book went to Colorado an invalid. He has given to the study of climate much time and careful thought prompted not only by scientific interests, but by strong personal motives. We have not yet perhaps the data on which to base positive conclusions, but from some knowledge of the Rocky Mountain regions from a somewhat extended observation of the effect of a residence in these regions upon chronic pulmonary disease, it seems quite certain that the conclusions of the author are in the main justified.

Dr. Denison sums up his study in a series of propositions. We print them in a condensed form. They have reference to consumption.

1. Cool, dry climates are desirable. These elements, coolness and dryness increase by elevation.

2. The Rocky Mountain region in coolness, dryness and sunshine meets the climatic indication.

3. The effect of di mnished atmospheric pressure from one sixth to one-fourth of that at sea level, is to promote the activity of the blood stream through the lungs.

4. A change to the Rocky Mountain climate in chronic lung troubles is favorable in proportion as it is resorted to early.

5. The stimulating effect of high altitudes upon heart and lungs is opposed to the idea of rest, usually thought necessary to repair, but is nevertheless an important agent in arresting chronic phthisis.

6. Tissue change should be stimulated, in at least the early stages of phthisis. Altitude hastens tissue change.

7. The curative influence of the Rocky Mountain climate is best shown in the early stages of chronic inflammatory and hæmorrhagic cases.

8. The contra-indications are: 1. Organic cardiac disease, especially if accompanied with abnormal activity. 2. Softening in acute cases with extensive deposits. 3. Chronic cases with extensive cavities. High temperature and advanced age should also probably be considered as contra-indications.

9. In most cases the change from low to high altitudes should be made gradually.

10. The altitude should vary in different cases according to condition, the range being from 2000 to 6500 feet. Severe or more advanced cases do not bear as well the higher altitudes.

11. In cases which improve there should be a prolonged residence—permanent if possible.

Dr. Denison has also prepared a chart for the examination of persons contemplating climatic change. It consists of a series of questions to be answered by the patient, as well as a complete summary of the medical facts to be investigated by the physician. The volume is enriched by a carefully prepared and executed map of the Eastern slope of the Rocky Mountains, giving the elevations, rain-fall, temperature, isothermal lines, humidity, force and direction of winds, roads, railroads, mineral springs, etc.

Upon the whole, we commend the book most cordially to physicians and others interested in the health resorts of what a few years ago was the "Far West," but which is now almost as near to Chicago as the Atlantic seaboard. J.

ARTICLE XIV.—ON COCCYGODYNIA. A Lecture Delivered in the Chicago Medical College, March 20th, 1880, by Edward W. Jenks, M.D., LL.D., Prof. of Medical and Surgical Diseases of Women, and of Clinical Gynæcology in the Chicago Medical College, etc. Reprinted from the Medical Record of N. Y., Vol. XVII, No. 16; April 17th, 1880.

This is a neatly printed pamphlet of fifteen pages, in which the author presents an excellent résumé of what is known concerning the very troublesome affection named in the title, with some additions of value from his own practice. He shows that two distinct morbid conditions have been included under the names coccygodynia, coxalgia, etc. One is an inflammation either traumatic or from rheumatism in the dorso-coccygeal articulation, the other a more obscure affection either of the nerves or fibrous structures attached to the coccyx. Of the first, many cases will recover by rest and appropriate medical treatment. Of the latter, a large majority of the cases are cured by surgical interference only. The lecture will well repay a careful perusal.

---

TREATMENT OF GONORRHŒA BY INJECTION OF CHLORAL.—Dr. Pasqua, head physician to the military hospital of Benghazi, indicates this method of treating gonorrhœa in a letter addressed to the *Bulletin Général de Therapeutique.* He employs a solution containing a gram and a half of chloral to 120 grams of vase water. Two injections a day retained some minutes have appeared sufficient. It produces at first a slight smarting, a sensation of prickling, which is followed in two or three minutes by an agreeable sensation of coolness. After the third or fourth day of treatment, the desire to urinate and the erections become less painful and frequent, the discharge diminishes, becomes more clear and limpid and ceases completely in eight or ten days. M. Pasqua has examined his patients at various intervals without finding any of the complications or accidents which often follow badly treated cases of gonorrhœa.—*La France Médicale*, May 12, 1880.

